# Dieffenbach’s Operation for Ununited Fracture

**Published:** 1854-07

**Authors:** 


*Dieffenbach's Operation for Ununited Fracture.*—Mr. Mackenzie exhibited (to the Medico-Chirurgical Society of Edinburgh) a thigh-bone on which he had practised Dieffenbach's operation for the cure of ununited fracture, the drilling of the ends of the bone and the insertion of an ivory peg.

The patient, a seaman, had fractured his thigh between two and three years before he was admitted into the hospital under Mr. M.'s care. He was treated in a public hospital in America, and he attributed the want of union to the limb not having been kept steady, the bandages confining the limb having been loosely applied.

The fracture was in the middle of the bone, and the lower fragment was retracted about an inch and a half on the inner side, and behind the upper. The limb was quite flexible at the seat of fracture, and was almost entirely useless as a means of support.

The ends of the bone were drilled with a small gouge worked by a carpenter's brace, and a peg, two and a half inches in length, was driven home with a hammer. The presence of the foreign body gave rise to such extensive inflammation and constitutional disturbance, that it was found necessary to remove it at the end of eight days. Profuse suppuration took place, undermining the whole deep textures of the thigh, and it seemed doubtful for some time whether the patient was not to sink from the effects of the operation. He rallied, however, and the suppuration gradually diminished. Firm osseous union took place, and at the end of some months the patient began to make use of the limb in walking. A sinus, however, continued to discharge matter at the seat of operation; and, after he had been walking for some weeks, he suffered from an attack of erythema of the limb; suppuration of the knee-joint followed, accompanied by hectic fever, and amputation was performed immediately above the seat of fracture.

The preparation showed the fractured ends firmly united by a large osseous deposit.

Mr. Mackenzie believed this was the only case in which Dieffenbach's method had been practised in Edinburgh. The result was not encouraging as regarded its further trial, at least in the thigh. A considerable amount of success had followed the practice, in ununited fractures of the leg and forearm, in the hands of the London hospital surgeons; but the only other case in which, as far as he could find, it had been attempted in the thigh, had proved equally unfortunate; he alluded to a case by Mr. Square of Plymouth, in which, as in the present case, it had been subsequently found necessary to perform amputation.—*Edinburg Monthly Journal of Medical Science.*

